# Association of different milk fat content with coronary artery disease and myocardial infarction risk: A Mendelian randomization study

**DOI:** 10.1371/journal.pone.0300513

**Published:** 2024-04-10

**Authors:** Jiacan Wu, Guanghong Tao, Hua Xiao

**Affiliations:** Department of Cardiology, The First Hospital of Chongqing Medical University, Chongqing, China; Neyshabur University of Medical Sciences, ISLAMIC REPUBLIC OF IRAN

## Abstract

**Background:**

Numerous observational studies have investigated on the correlation of whole, semi-skimmed, and skimmed milk with coronary artery disease (CAD) and myocardial infarction (MI) risk; However, no consensus has been reached and evidence on any causal links between these exposures and outcomes remains unclear. This study aimed to conduct univariate and multivariate Mendelian randomization (MR) analyses, using publicly released genome-wide association study summary statistics (GWAS) from the IEU GWAS database, to ascertain the causal association of milk with various fat content with CAD and MI risk.

**Methods:**

For the exposure data, 29, 15, and 30 single-nucleotide polymorphisms for whole milk, semi-skimmed milk, and skimmed milk, respectively, obtained from 360,806 Europeans, were used as instrumental variables. CAD and MI comprised 141,217 and 395,795 samples, respectively. We used inverse variance weighted (IVW), weighted median, MR-Egger regression, and MR Pleiotropy Residual Sum and Outlier analyses to determine whether pleiotropy and heterogeneity could skew the MR results. Sensitivity tests were conducted to verify the robustness of the results.

**Results:**

After adjusting for false discovery rates (FDR), we discovered proof that skimmed milk intake is a genetically predicted risk factor for CAD (odds ratio [OR] = 5.302; 95% confidence interval [CI] 2.261–12.432; *P* < 0.001; FDR-corrected *P* < 0.001) and MI (OR = 2.287; 95% CI 1.218–4.300; *P* = 0.010; FDR-corrected *P* = 0.009). Most sensitivity assessments yielded valid results. Multivariable MR for CAD and MI produced results consistent with those obtained using the IVW method. There was no causal relationship between whole or semi-skimmed milk, and CAD or MI.

**Conclusion:**

Our findings indicate that the consumption of skimmed milk may increase the risk of CAD and MI. This evidence may help inform dietary recommendations for preventing cardiovascular disease. Further studies are required to elucidate the underlying mechanisms.

## Introduction

Coronary artery disease (CAD) and myocardial infarction (MI) risk are the two main causes of mortality in developed countries [1, 2]. Milk is an essential part of the western diet because it is rich in nutrients. It is composed of approximately 30% unsaturated fats and 70% saturated fats [3, 4]. Milk can be approximately categorized into three types: whole milk (3.5%), semi-skimmed milk (1.6%), and skimmed milk (≤0.5%) based on fat content [3, 5, 6]. The incorporation of milk as a modifiable dietary component has been suggested to help prevent cardiovascular diseases (CVD) [7, 8]. Previous studies have investigated the effects of whole, semi-skimmed, and skimmed milk on CVD risk [9, 10]. Whole milk may increase the plasma levels of low-density lipoprotein (LDL) cholesterol, a major risk factor for CAD [11, 12]. Therefore, low-fat products are advised by both the American and European Heart Associations to lower the risk of CVD [13, 14].

In contrast, the Multi-Ethnic Study of Atherosclerosis suggested lower coronary artery calcification prevalence and progression rates in whole milk consumers than in non-consumers [15]. Based on data from a large multinational Prospective Urban Rural Epidemiology study [16], consumption of whole-fat dairy is linked to a reduced risk of CVD, including acute MI and associated mortality in the general public. This suggests a positive impact of whole-fat dairy foods on health. A prospective study by Farvid et al. demonstrated that milk and dairy intake were not associated with coronary heart disease (CHD) [17]. A study by Kristin et al. reached similar conclusions [18].

Overall, the evidence linking the consumption of different milk types to CVD is inconclusive and further prospective, large-scale, and long-term randomized controlled trials are required to evaluate this relationship [19]. Given the conflicting findings of previous studies, confounding bias in many observational studies, and reverse causation in prospective cohort studies, we applied a two-sample mendelian randomization (MR) to comprehensively investigate the potential causal role of whole, semi-skimmed, and skimmed milk consumption, in the development of CAD and MI.

MR uses genetic data to assess potential causal relationships between exposures and outcomes [20]. Through random assignment of single-nucleotide polymorphisms (SNPs) during conception, MR can help minimize the effects of residual confounding [21]. Prior MR studies have investigated the causal link between milk intake and CVDs [22–24], but the association between milk assumption with different fat content and CAD and MI has not been reported previously.

## Materials and methods

### Study design

**Fig 1:**
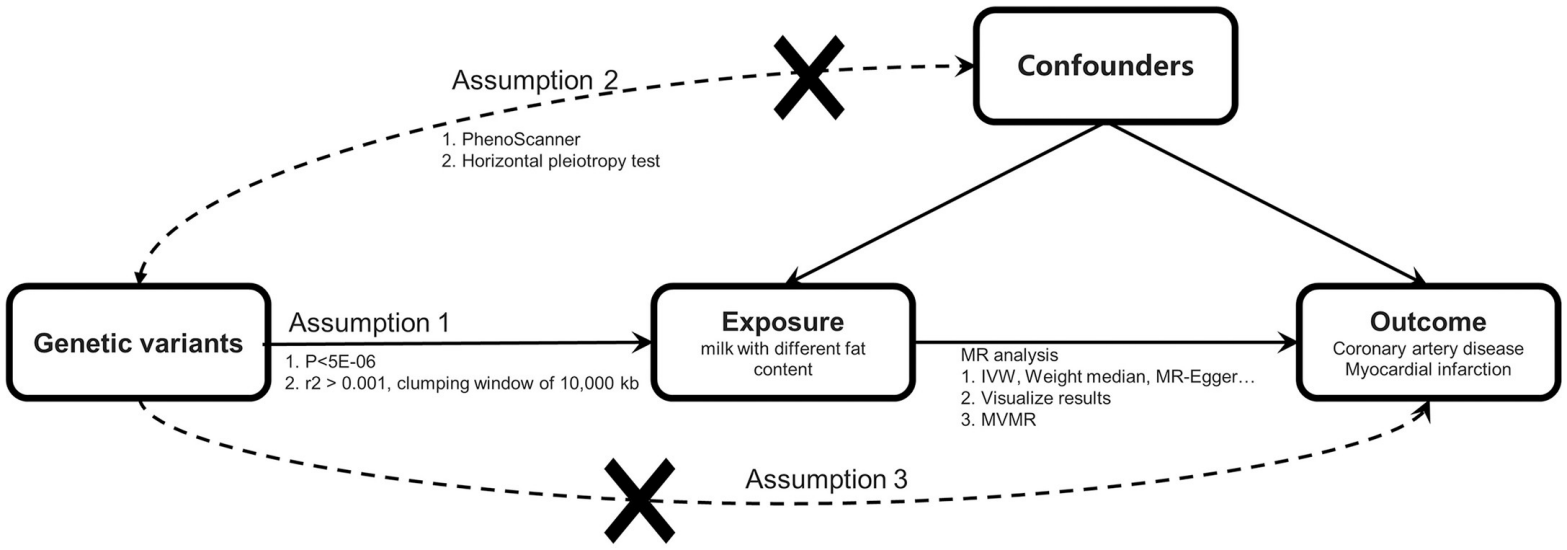
Study design of MR. Three core assumptions were as follows: (1) Relevance assumption, the genetic variants must be associated with exposure (milk with different fat content); (2) Independence assumption, the genetic variants should not be associated with confounders; (3) Exclusion restriction, the genetic variants must influence outcome (coronary artery disease and myocardial infarction) only via exposure.

We conducted MR to infer the causal relationship between exposure (whole, semi-skimmed, and skimmed milk) and outcomes (CAD and MI) using genetic variants strongly associated with exposure as instrumental variable (IV). The MR design relies on three crucial assumptions [25] (Fig 1) regarding genetic variation:

1. The genetic variants have a strong association with the exposure;
2. The genetic variants are independent of potential confounding factors;
3. The genetic variants influence outcomes solely through exposure factors.

### Data sources

We selected SNPs as IVs for all exposures (whole, semi-skimmed, and skimmed milk) and outcomes (CAD and MI) from the IEU GWAS database, which is a database of genetic associations from GWAS summary datasets [26]. SNPs related to milk type were identified in a GWAS dataset of European populations by the UK Biobank, which contains data of over 500,000 participants aged 40–69 years recruited across the UK from 2006 to 2010. Data on the milk type used (n = 360806) were obtained using an electronic questionnaire asking, “what type of milk do you mainly use?”, with responses including: “whole,” “half,” “skim,” “soya,” “other types of milk,” “never/rarely drink milk,” “don’t know,” and “don’t want to answer.” We extracted data for whole milk (n = 22,902), semi-skimmed milk (n = 234,870), and skimmed milk (n = 74,087). GWAS IDs used were ukb-d-1418-1, ukb-d-1418-2, and ukb-d-1418-3.

The primary outcomes were CAD and MI. To mitigate the population stratification bias effects, most of our data were derived from individuals of European descent. CAD was derived from the CARDIoGRAMplusC4D Consortium (n = 42,096 cases and 361 controls) [27], and MI was derived from Jana et al. (n = 14,825 cases and 44,000 controls) [28]. GWAS IDs are ebi-a-GCST003116 and ebi-a-GCST011365.

### Ethics statement

All participants provided written informed consent to be involved in the original study, which reports the relevant details. As this study involved analysis of publicly available GWAS datasets, additional ethical approval was not required.

### Instrument variable selection

We selected ideal SNPs in four steps. First, only SNPs that were strongly related to milk types were included. Since few (≤4) SNPs were significant at the genome-wide significance level (*P* < 5e-08) for milk type, we adopted a larger significance threshold (*P* < 5e-06) for IV selection [29]. Secondly, we excluded SNPs with a linkage disequilibrium [30] (r2 > 0.001 and clumping window of 10,000 kb) to confirm the independence of the selected genetic variants. Third, we calculated the F-statistic to assess the strength of individual SNPs, considering sufficient at an F-value of >10 [20]. Finally, before conducting the MR analysis, we ensured that the effect of an SNP on exposure and outcome was relative to the same allele by harmonizing them, removing duplicates, and excluding palindromic SNPs (A/T or C/G) from the analysis.

### Statistical analyses

We applied the inverse variance weighted (IVW) as the main analysis method, utilizing the fixed-effects model to assess the causal relationship between different milk types consumptions and CAD and MI risk. Then, the MR-Egger regression and weighted median method were used to complement the sensitivity analysis. The weighted median can provide valid estimates if at least 50% of the information in the analysis comes from SNPs as valid IVs [31]; the MR-Egger method can estimate the horizontal pleiotropy of selected IVs [32]. Nest, we conducted MR Pleiotropy Residual Sum and Outlier (MR-PRESSO) analysis to test the horizontal pleiotropy and obtained a corrected association result after removing the potential outlier SNPs [33]. In addition, the heterogeneity of IVs was assessed using Cochran’s Q test. In case of observed heterogeneity, an IVW analysis was conducted using a random-effects model. Finally, we used leave-one-out method to assess sensitive SNPs that influence causal associations. Forest and scatter plots were used in an addition to the sensitivity analysis. Moreover, we retrieved the potential secondary phenotypes of each IV from PhenoScanner (http://www.phenoscanner.medschl.cam.ac.uk/) [34], selected SNPs that were exclusively associated with exposures to ensure that the SNPs were not related to any potential covariates, and conducted MR analysis as a supplementary sensitivity analysis to enhance the robustness of the results.

We further subjected meaningful results from the univariable MR to multivariable MR analysis to assess the robustness of our findings. In multivariable MR, adjustments were made for 14 potential confounding factors, including smoking, obesity, hypertension (HT), diabetes (DM), hyperlipidemia, atrial fibrillation, heart failure, chronic kidney disease, peripheral arterial disease, depression, vitamin D deficiency, serum uric acid levels, plasma levels of LDL, and life stress [35–38]. Detailed information regarding the source of the confounding factors in the GWAS data can be found in S10 Table in S1 File. Similar to the univariable MR, we selected independent SNPs with p-values of < 5e-06 and LD r2 of > 0.001, with the clumping distance of 1000kb. The analysis was performed using the IVW, weighted median, and MR-Egger methods. Multiple comparisons were performed for milk with different fat content and CAD and MI, and we conducted FDR correction with p.adjust() function in R. All calculations were performed through the “TwoSampleMR,” “MR-PRESSO,” and “MendelianRandomization” packages in R software (Vision 4.2.2).

## Result

We identified 29 SNPs strongly associated with skimmed milk, 15 SNPs associated with semi-skimmed milk, and 30 SNPs associated with whole milk. The F-values of all genetic instruments were greater than 10, suggesting the absence of weak IVs (S1-S3 Tables in S1 File).

### Univariable MR

#### Skimmed milk

**Fig 2:**
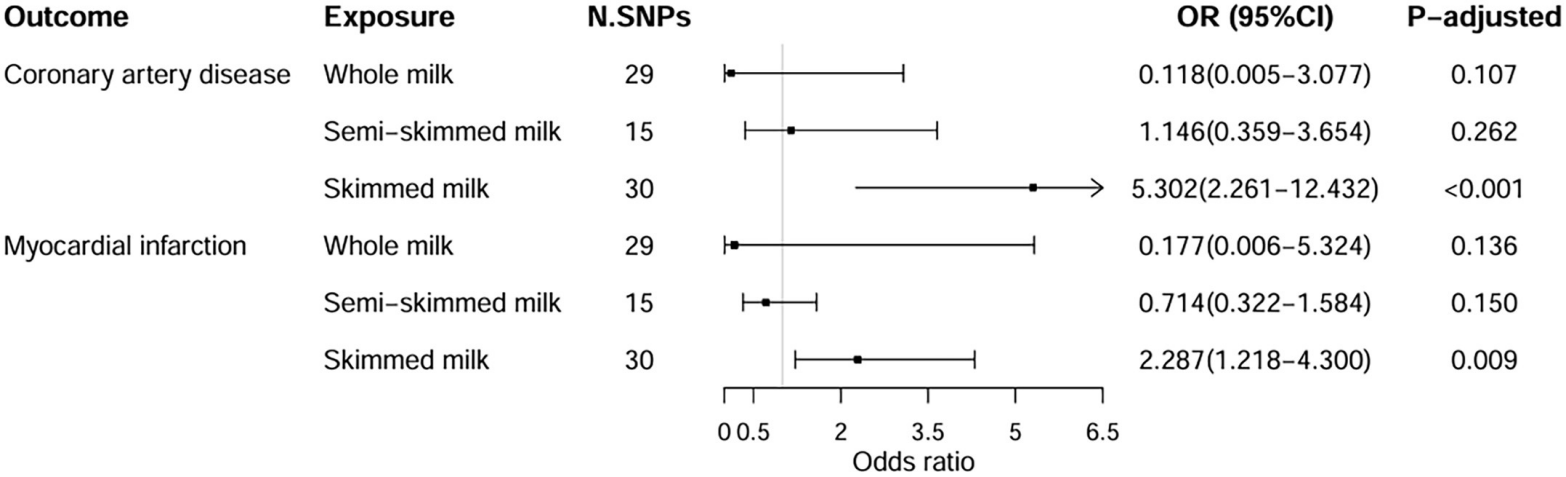
Associations of whole, semi-skimmed, and skimmed milk with coronary artery disease and myocardial infarction. CI, confidence interval; OR, odds ratio; SNP, single-nucleotide polymorphism; *P*_-adjusted_, FDR-corrected *P*.

The IVW method showed that skimmed milk consumption was a genetically predicted risk factor for CAD (odds ratio [OR] = 5.302; 95% confidence interval [CI] 2.261–12.432; *P*<0.001; FDR-corrected *P*<0.001) and MI (OR = 2.287; 95% CI 1.218–4.300); *P* = 0.010; FDR-corrected *P* = 0.009) (Fig 2). The ORs obtained from the MR-Egger regression and weighted median methods aligned with the IVW results, albeit with a slightly lower precision (S4 Table in S1 File). No evidence of unbalanced horizontal pleiotropy or outlier SNPs in either outcome was found in the MR-PRESSO analysis (S5 Table in S1 File). In addition, horizontal pleiotropy was not detected using MR-Egger intercept testing. Cochran’s Q statistic did not reveal any evidence of heterogeneity among the selected IVs (S6 Table in S1 File).

The leave-one-out sensitivity analysis is shown in S1 and S2 Figs in S2 File. It revealed that the overall estimates were unaffected by any individual SNP. Forest and scatter plot can be seen in S3-S6 Figs in S2 File. To ensure that the SNPs were not related to potential covariates, we searched all SNPs in the PhenoScanner database and deleted each potential SNPs associated with the main traits that may create pleiotropy, such as smoking or cigarette use, obesity, HT, and DM (S7 Table in S1 File). We re-conducted the MR analysis, and the results were consistent with those of univariable MR (S8 and S9 Tables in S1 File).

#### Whole milk and semi-skimmed milk

IVW analysis did not reveal a causal association between whole milk with CAD (OR = 0.118; 95% CI 0.005–3.077; FDR-corrected *P* = 0.107;) or MI (OR = 0.177; 95% CI 0.006–5.324; FDR-corrected *P* = 0.136) (Fig 2). The MR-Egger analysis and weighted median method broadly showed similar effects to IVW (S4 Table in S1 File). Although MR-Egger intercept testing found no evidence of horizontal pleiotropy, MR-PRESSO showed the opposite result (*P*-value global test < 0.05) and identified two outlier SNPs for CAD and four outlier SNPs for MI (S5 and S6 Tables in S1 File). Removing the outlier SNPs did not change the results. Cochran’s Q statistics demonstrated the presence of heterogeneity in the included IVs, so we using an IVW analysis under a random-effects model (S6 Table in S1 File). The leave-one-out analysis can be attached in S7 and S8 Figs in S2 File, and detailed forest and scatter plots are shown in S9-S12 Figs in S2 File.

MR analysis did not suggest any genetically determined semi-skimmed milk consumption-related risk and CAD (OR = 1.146; 95% CI 0.359–3.654; FDR-corrected *P* = 0.262) or MI (OR = 0.714; 95% CI 0.322–1.584; FDR-corrected *P* = 0.150) (Fig 2). The OR values derived from the MR-Egger analysis and weighted median method were similar, showing directional consistency between the two CVDs (S4 Table in S1 File). Similarly, the MR-Egger method and MR-PRESSO analysis did not reveal horizontal pleiotropy across either outcome (S5 and S6 Tables in S1 File). No outlier SNP was found in the MR-PRESSO analysis. Additionally, no evidence of heterogeneity for the selected IVs across outcomes was observed (S6 Table in S1 File). The leave-one-out plot is in presented in S13 and S14 Figs in S2 File, and the forest and scatter plots are presented in S15-S18 Figs in S2 File. By querying the potential secondary phenotypes of each SNP and reconducting the MR after excluding SNPs, which could introduce confounding factors, we found that additional analyses indicated no causal association between whole or semi-skimmed milk consumption and the risk of CAD and MI (S8 and S9 Tables in S1 File).

### Multivariable MR

To evaluate the direct causal effects of skimmed milk on the risk of CAD and MI, we further conducted a multivariable MR analysis. The effect of genetically predicted skimmed consumption-related risk on CAD and MI was further supported by the consistent direction and magnitude derived from distinct MR models after accounting for 14 confounding factors, post-FDR adjustments. Detailed information regarding the sources of the relevant exposures data can be found in S11 and S12 Tables in S1 File.

## Discussion

As far as we comprehend, this is the first MR study examining the association between different fat content in milk and the risk of cardiovascular diseases. In this study, univariable MR analysis revealed a positive association between consumption of skimmed milk and an elevated risk of CAD and MI. In contrast, no causal association was found between whole and semi-skimmed milk consumption and CAD and MI risk. Multivariable MR analysis yielded the consistent results, confirming the stability of the primary results.

Our results suggest an adverse effect of skimmed milk consumption on CAD and MI risk, which is supported by the findings of some previous studies. A prospective cohort study including 1,759 participants with 16.2 years of follow-up reported that consumption of low-fat and skimmed dairy products (but not whole milk) was associated with a higher incidence of CHD [39]. Further, a recent univariable MR study on the association between the types of milk and HT also indicated an adverse effect of skimmed milk [40]. In the present study, we conducted multivariable MR analysis based on univariate MR findings, adjusting for various confounding factors, and showed adverse direct causal effect of skimmed milk consumption on CAD and MI risk. It suggests that we may have overly focused on the adverse impact of milk fat content. Lauric acid, the main saturated fatty acid in milk, will elevate high-density lipoprotein cholesterol (HDL), and stearic acid can reduce the ratio of total cholesterol to HDL and play a protective role [3]. What’s more, milk is rich in various nutrients, which should not be overlooked in favor of focusing on fat content. For instance, vitamin D deficiency has been linked to the risk of CAD and MI [41, 42], and reduced fat-soluble vitamin D content in skimmed milk may contribute to an increased risk of CAD and MI.

Our findings differ from those of a previous case-control study by Khatun et al. [43], who compared detailed-dietary patterns in 105 patients with CHD and 105 individuals without CHD and suggested low-fat milk consumption as a protective factor against CHD. Another cross-sectional study observed that individuals with low-fat milk patterns (low-fat and skimmed milk) may have a reduced risk of CHD [44]. These studies had small sample sizes and were subject to recall bias. However, an 11.6-year prospective cohort study showed no association between milk intake and MI, regardless of milk fat content [45]. This previous study only focused on female participants, whereas our study did not include sex-stratified analyses. Further studies are required to account for any sex-based differences in this context. A recent systematic review indicated that the consumption of milk with varying fat content is not associated with CVD [46], but due to the paucity of studies and the low quality of data involved [47], the effects of milk consumption on CVD remain unclear. Despite the new insights provided by our study, more research is needed in the future to further explore the relationship between milk with different fat content and CAD and MI risk, as well as the underlying mechanisms.

The main strength of our study is the application of a two-sample MR design. In addition, our data is derived from a large sample in the GWAS database. Moreover, we conducted FDR correction on the results and carried out various sensitivity analyses and multivariable MR analyses to ensure the robustness of the results. However, this study has some limitations. First, this study focused on European populations, with most participants being of European descent; therefore, generalizability of these findings to other populations should proceed with caution, as some evidence suggests population-level differences in factors such as milk consumption capacity and dietary habits. Second, it was difficult to exclude potential directional polymorphisms; therefore, we performed MR-PRESSO to exclude outliers, and no evidence of horizontal pleiotropy was observed in the sensitivity analysis. Finally, the lack of exploration into the relationship between milk intake and cardiovascular diseases, along with the absence of subgroup analyses for different genders and age groups, hinders our further analysis. While our study provides insights into the selection of milk with varying fat content, future research should delve deeper to investigate the most suitable types and amounts of milk intake for diverse populations.

## Conclusion

In summary, this study suggests a genetic vulnerability for CAD and MI linked to skimmed milk consumption. The management of skimmed milk intake may help to reduce the risk of CAD and MI. This evidence should be considered preliminary and further large-scale long-term prospective cohort studies are required to validate it. Further exploration of the underlying mechanisms is essential.

## Supporting information

S1 File S1-S12 Tables are included in the file. (ZIP)

S2 File S1-S18 Figs are included in the file. (ZIP)
