# Supplementary material for: Association of different milk fat content with coronary artery disease and myocardial infarction risk: A Mendelian randomization study
Source: PLoS One. 2024 Apr 10;19(4):e0300513. doi: 10.1371/journal.pone.0300513 (PMC11006182; doi:10.1371/journal.pone.0300513)
Supplement: S2 File — (ZIP) [file pone.0300513.s002.zip › S2 File. Supporting Figs.docx]

**Supporting Figures**

**Supplementary Fig 1.** Leave-one-out sensitivity analysis for CAD using primary genetic instruments;


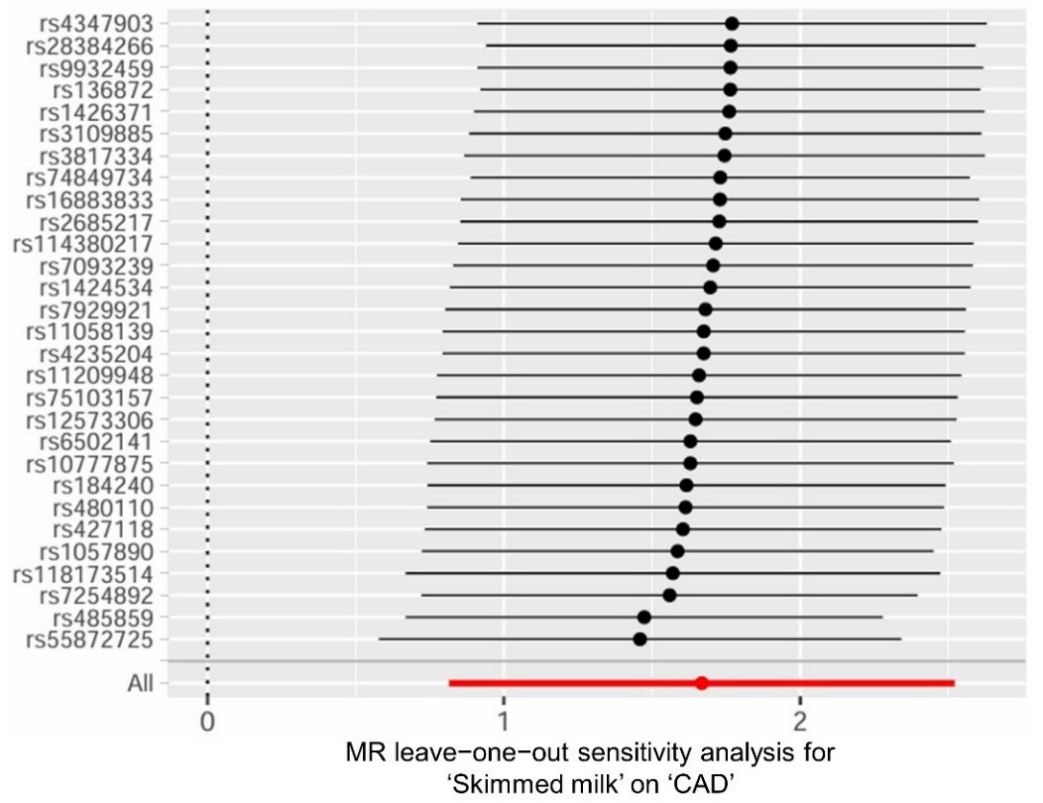


**Supplementary Fig 2.** Leave-one-out sensitivity analysis for MI using primary genetic instruments;


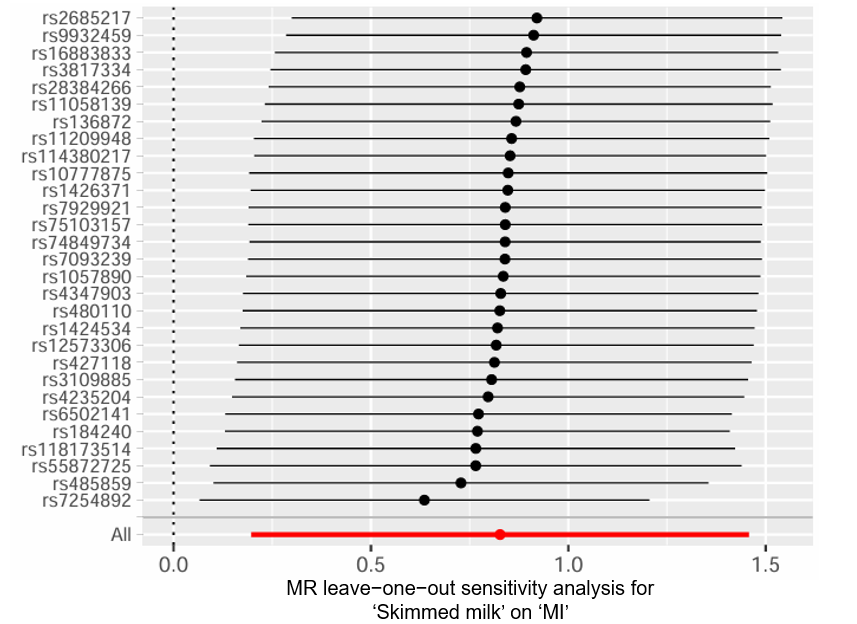


**Supplementary Fig 3.** The forest plot for the causal effects of skimmed milk-associated SNPs on CAD;

**
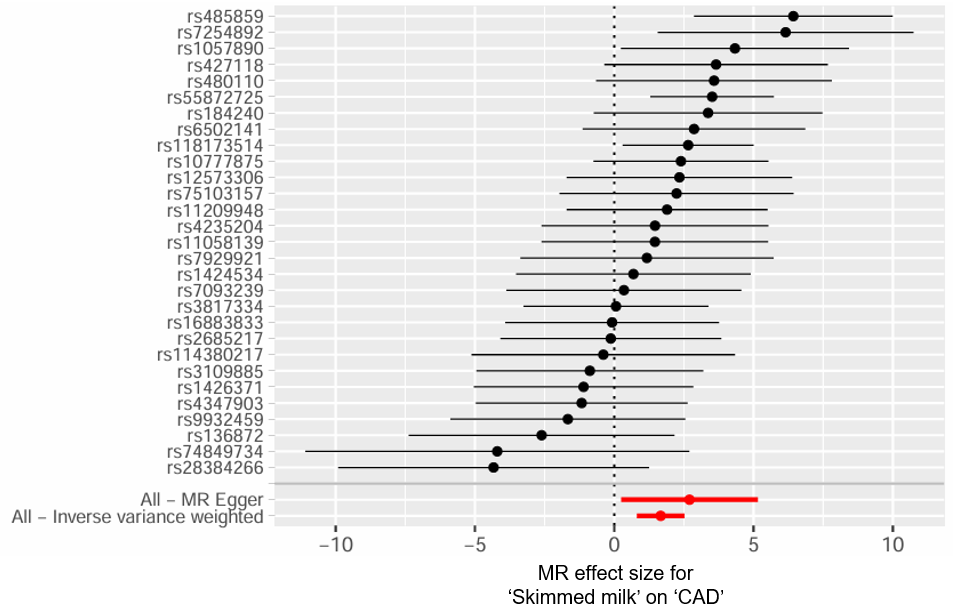
**

**Supplementary Fig 4.** The forest plot for the causal effects of skimmed milk-associated SNPs on MI;

**
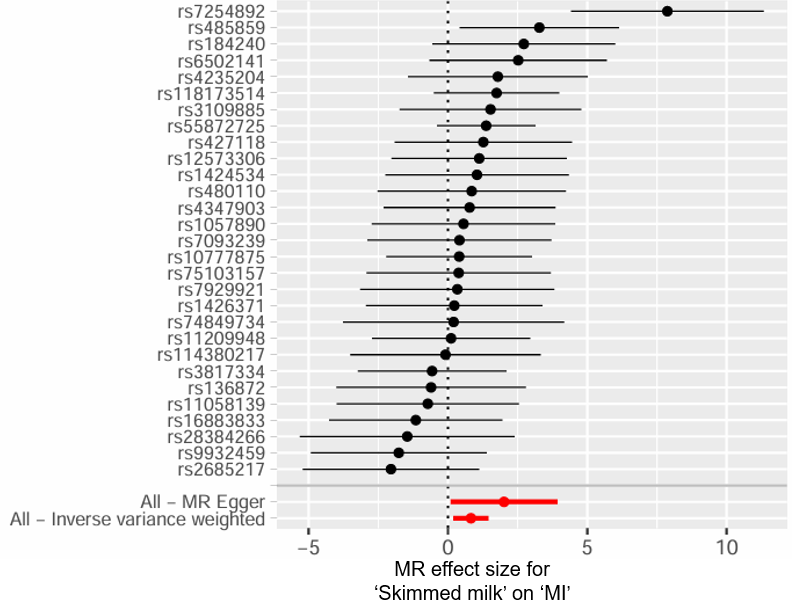
**

**Supplementary Fig 5.** Scatter plot of genetic associations comparing skimmed milk to the genetic associations with CAD**;**

**
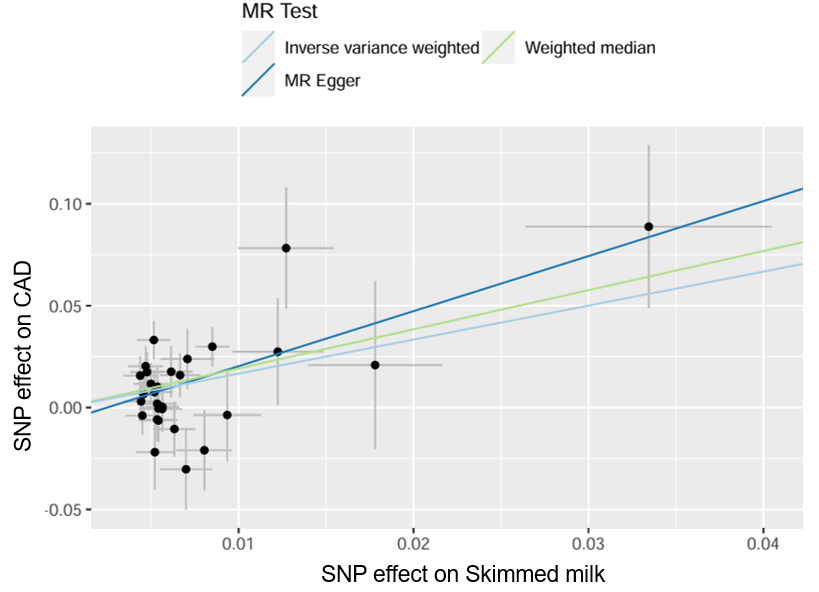
**

**Supplementary Fig 6.** Scatter plot of genetic associations comparing skimmed milk to the genetic associations with MI**;**

**
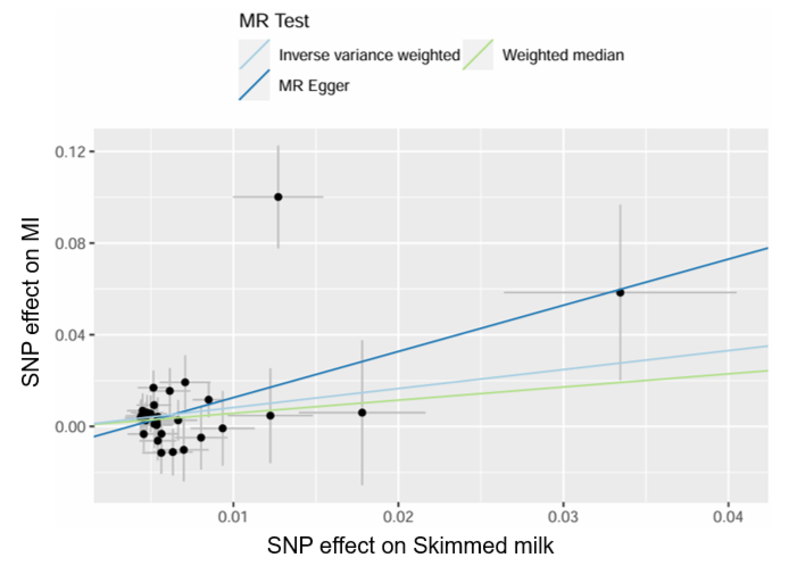
**

**Supplementary Fig 7.** Leave-one-out sensitivity analysis for CAD using primary genetic instruments;

**
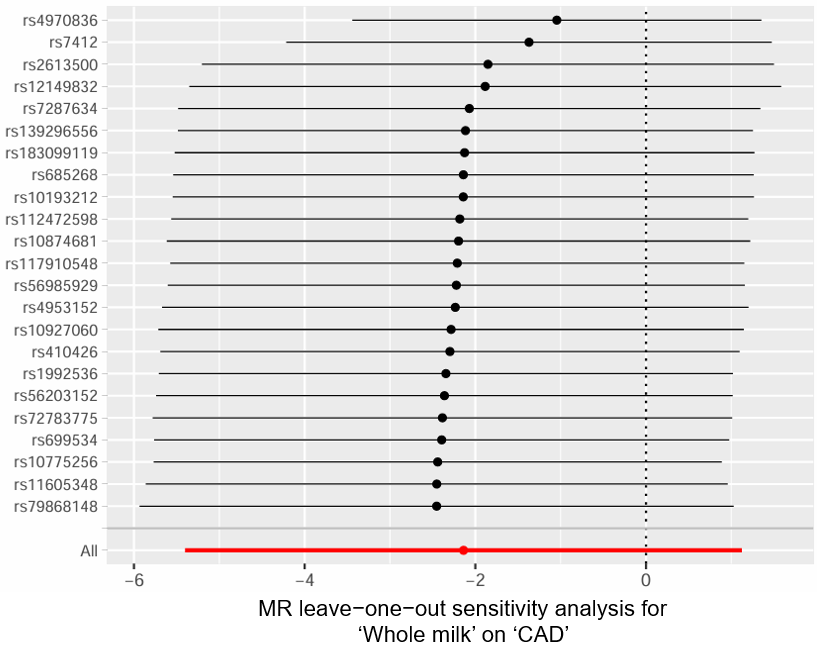
**

**Supplementary Fig 8.** Leave-one-out sensitivity analysis for MI using primary genetic instruments;

**
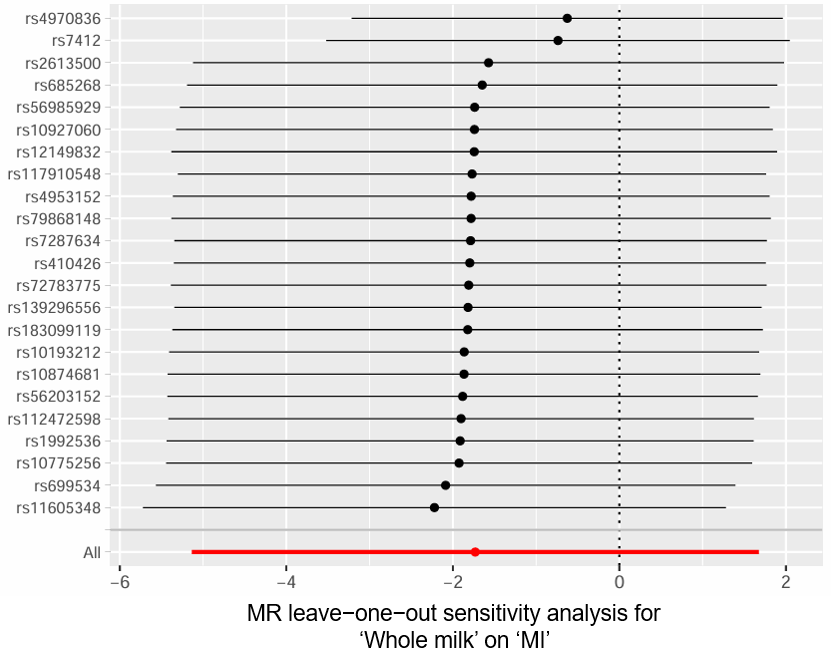
**

**Supplementary Fig 9.** The forest plot for the causal effects of whole milk-associated SNPs on CAD;

**
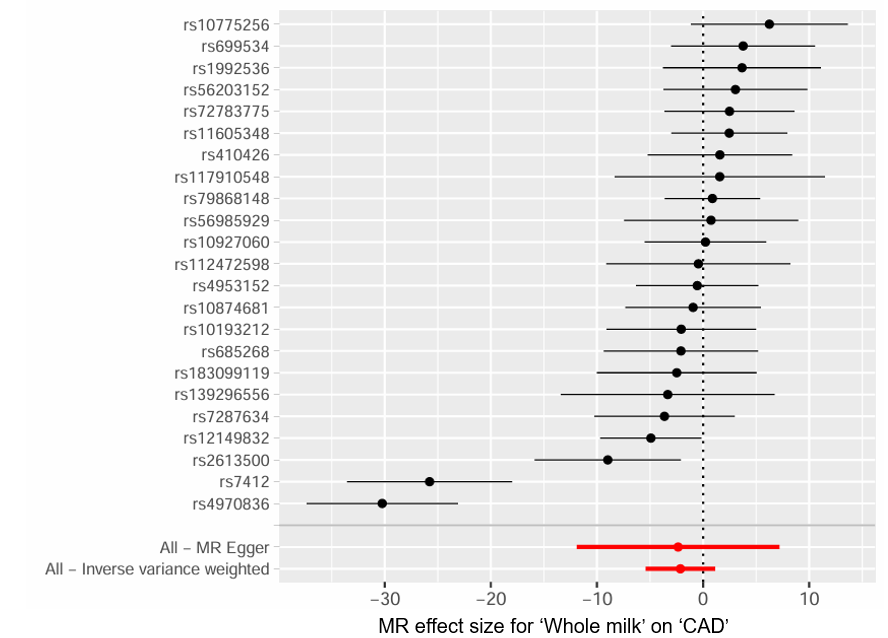
**

**Supplementary Fig 10.** The forest plot for the causal effects of whole milk-associated SNPs on MI;

**
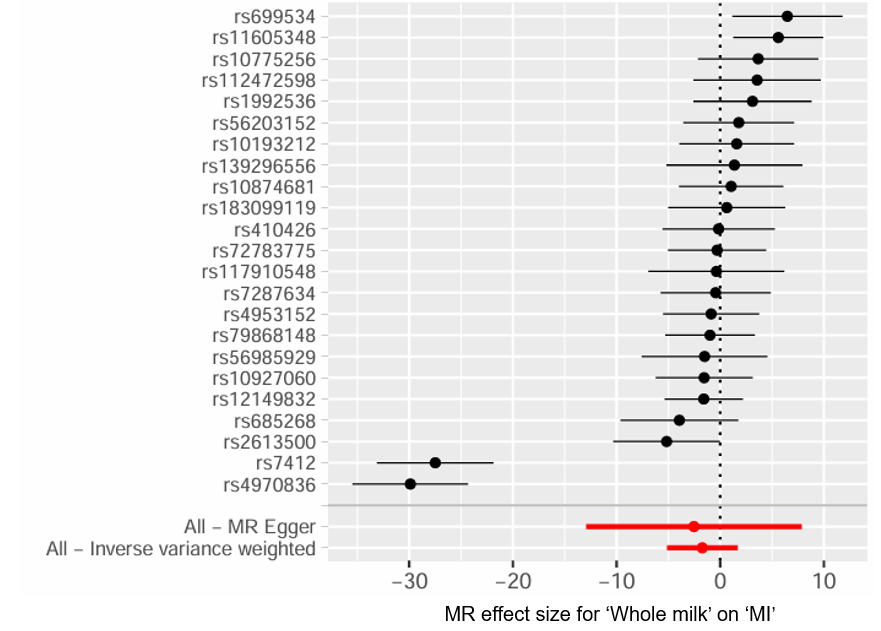
**

**Supplementary Fig 11.** Scatter plot of genetic associations comparing whole milk to the genetic associations with CAD;

**
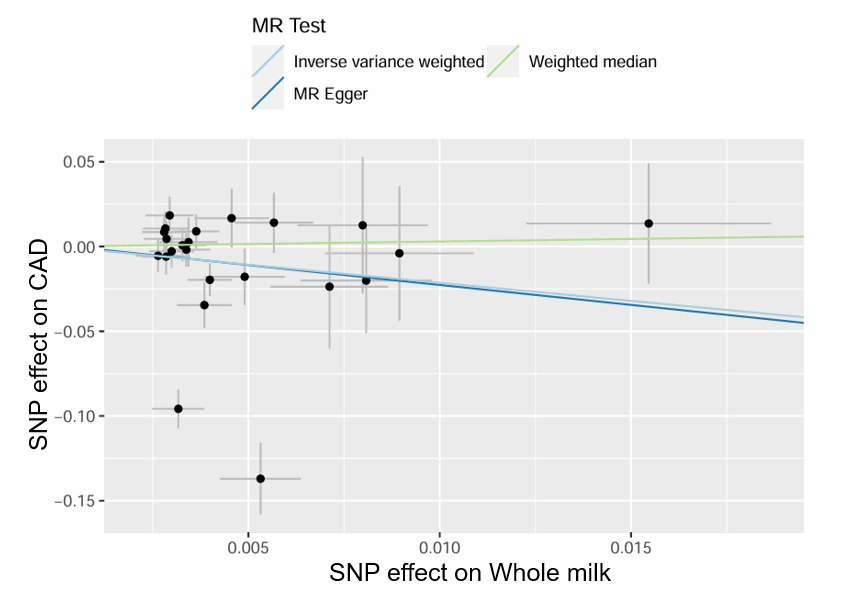
**

**Supplementary Fig 12.** Scatter plot of genetic associations comparing whole milk to the genetic associations with MI;

**
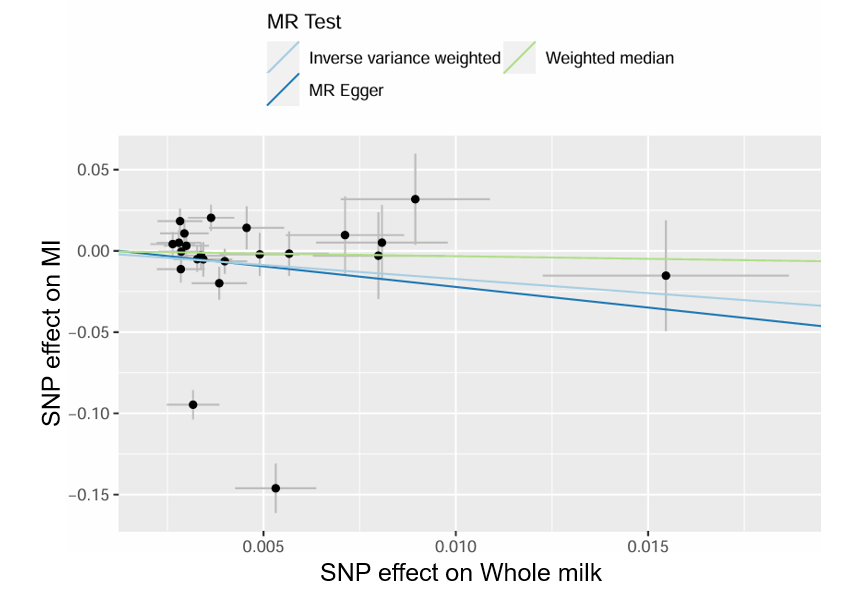
**

**Supplementary Fig 13.** Leave-one-out sensitivity analysis for CAD using primary genetic instruments;

**
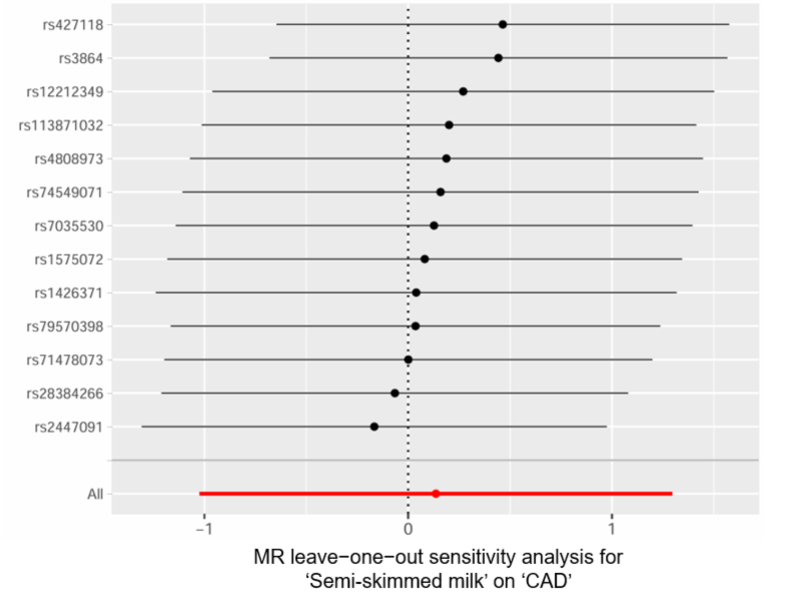
**

**Supplementary Fig 14.** Leave-one-out sensitivity analysis for MI using primary genetic instruments;


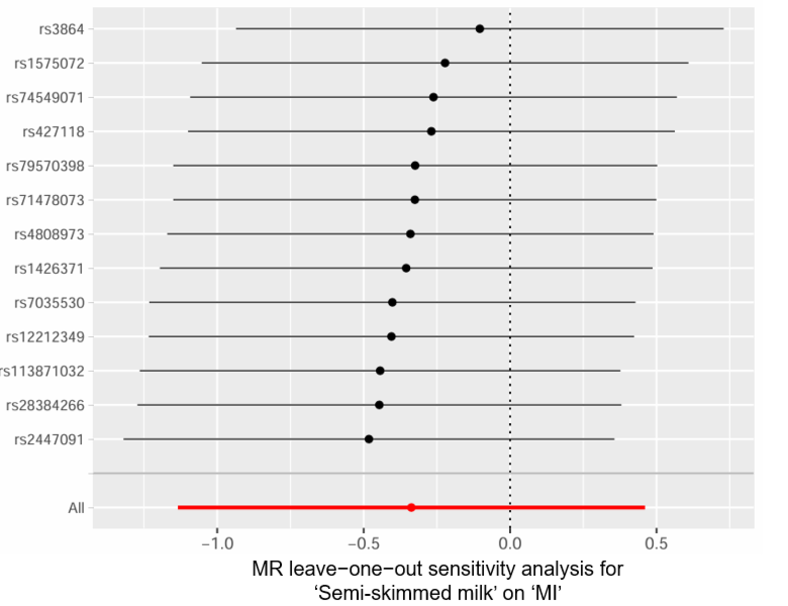


**Supplementary Fig 15.** The forest plot for the causal effects of semi-skimmed milk-associated SNPs on CAD;


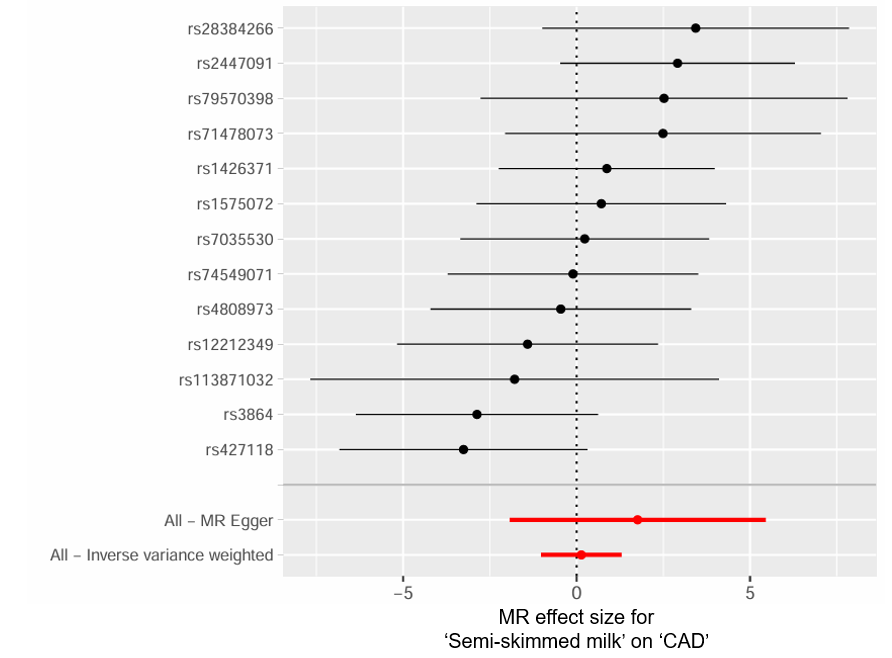


**Supplementary Fig 16.** The forest plot for the causal effects of semi-skimmed milk-associated SNPs on MI;

**
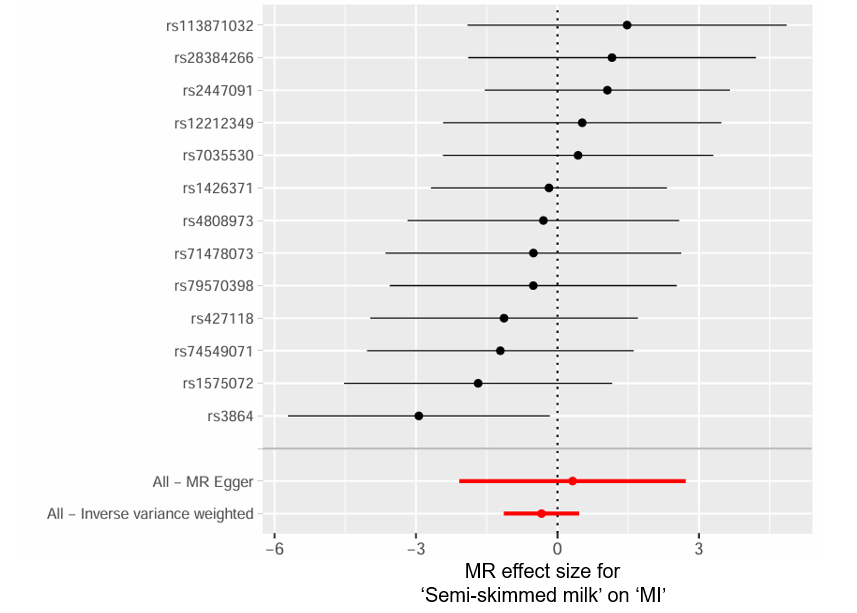
**

**Supplementary Fig 17.** Scatter plot of genetic associations comparing semi-skimmed milk to the genetic associations with CAD;

**
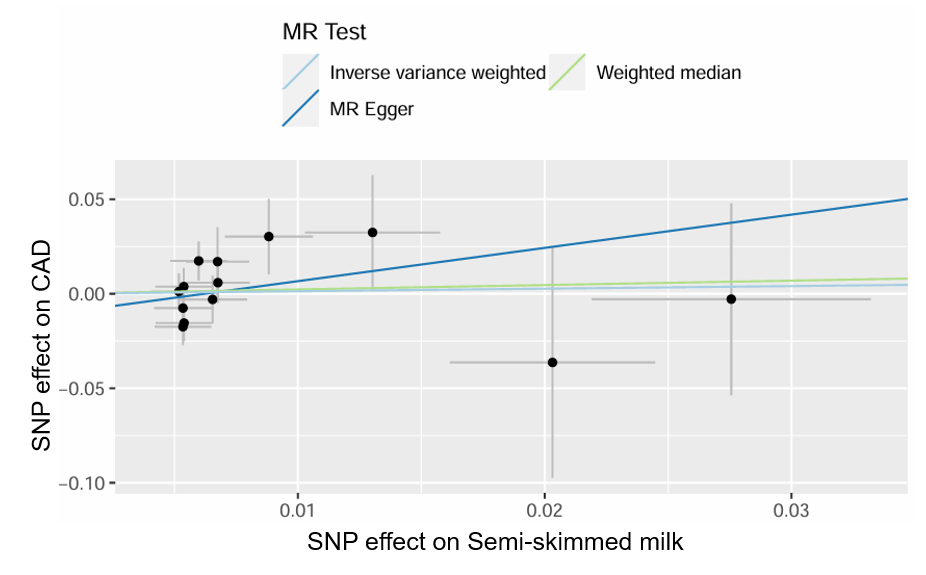
**

**Supplementary Fig 18.** Scatter plot of genetic associations comparing semi-skimmed milk to the genetic associations with MI;

**
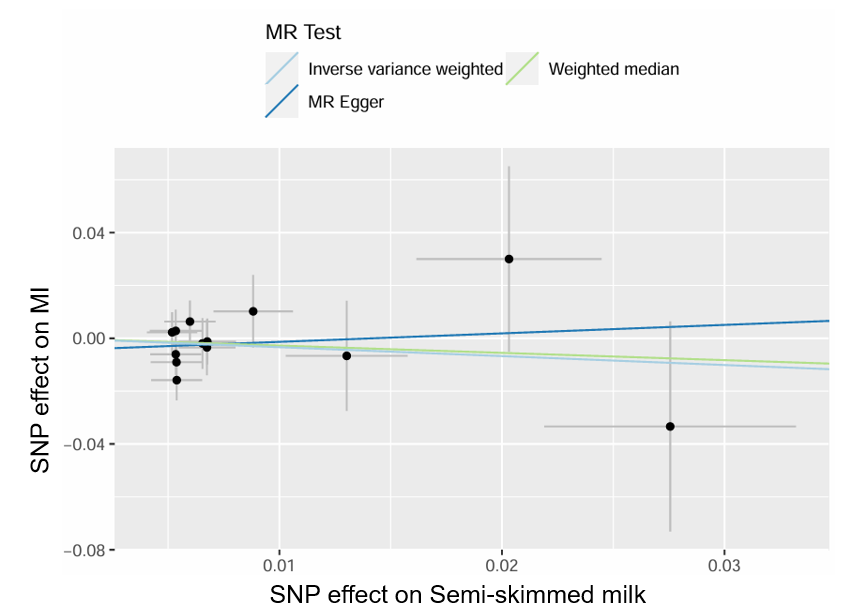
**
